# Evaluation of Dose Dependent Maternal Exposure to Bisphenol A on Thyroid Functions in Newborns

**DOI:** 10.3390/jcm7060119

**Published:** 2018-05-23

**Authors:** Burcin Sanlidag, Ceyhun Dalkan, Osman Yetkin, Nerin N. Bahçeciler

**Affiliations:** 1 Pediatrics, Faculty of Medicine, Near East University, 99138 Nicosia, Cyprus; dalkanc@yahoo.com (C.D.); nerin74@gmail.com (N.B.); 2 Department of Medical Biochemistry, Faculty of Medicine, Near East University, 99138 Nicosia, Cyprus; osman.cyp@gmail.com

**Keywords:** Bishenol A, thyroid hormone, newborn

## Abstract

Bisphenol A (BPA) is an endocrine-disrupting chemical compound that is mainly used in industrial products as packaging and plastics. It usually transmits to humans via oral route from food-contact material. BPA has demonstrated to be found in body fluids with a higher amount of fetal tissues due to bio-accumulation. Although it has been reported to affect the endocrine system, results on thyroid functions of newborns are conflicting. The aim of the present study is to demonstrate the effect of different levels of BPA in cord blood on the thyroid functions of newborns, according to gender. Methods: The study population included 88 newborns. The BPA levels, Thyroid stimulating hormone (TSH) and free thyroxine (fT4) levels of cord blood were measured. In addition, SPINA-GT (thyroid’ incretory capasity), TSH Index (TSHI), standardized TSHI (sTSHI) were calculated and demographic characteristics of participants were noted. Results: The mean of cord blood BPA was 4.934 ± 2.33 ng/mL. When evaluated according to quantiles of BPA, no association was found between BPA and thyroid hormone levels, as well as, SPINA-GT, TSHI, sTSHI in both genders. Conclusion: Although BPA has been shown to contaminate cord blood, no significant effect was detected on thyroid hormones, SPINA-GT, TSHI and sTSHI. Further investigations with larger study populations are warranted.

## 1. Introduction

Bisphenol A (BPA) is an industrial product mainly used in the manufacturing of food packaging, including polycarbonate plastics, epoxy resins lining metal cans and in dental sealants [1]. BPA does not bound chemically to the materials applied, therefore it can easily transfer to indoor dust, food, water and ambient air [2,3]. Exposure of BPA-containing products to increased temperature, has been reported to increase the content of BPA in foods or products [4]. Although the main way of transmission to humans is orally from food-contact materials, it has also been recorded to be transmitted by inhalation and through dermal absorption [5,6,7]. In humans, elimination half-life of BPA is 6 h and is mainly stored in adipose tissue [8]. In general, BPA is accepted as an endocrine-disrupting chemical compound in humans [9,10].

More importantly, BPA has been demonstrated to be found in the human umbilical cord, amniotic fluid, urine samples and maternal milk [11,12]. Poimenova et al. demonstrated a high bio-accumulation of BPA in fetal tissue when compared with maternal tissues [13]. This accumulation of BPA in humans and neonates has been identified as having a major impact on the endocrine system [14,15].

Rodent studies revealed that early life BPA exposure resulted in abnormalities in the development of the pituitary-thyroid axis [16,17]. The effect of BPA on thyroid functions during the critical developmental stage of the fetus may adversely affect neurodevelopmental outcomes [18,19].

BPA may interfere with thyroid hormone (TH) action by interacting directly with the TH receptor, affecting the normal delivery of the TH to target cells, or altering the metabolism of thyroid hormones (Ths) including thyroid stimulating hormone (TSH), free and total thyroxine, or FT3 and TT3 [20]. In vivo experiments suggested that BPA administration may induce maternofetal hypothyroidism via thyroid dysgenesis and dyshormonogenesis [21].

Few studies have examined the effect of prenatal BPA exposure on thyroid functions of newborns. Prenatal urinary BPA concentrations were found to be associated with reduced TSH in male newborns in a pregnancy cohort study from California [14]. In another study, a 10-fold increase in mean of urinary BPA of mothers was associated with lower cord TSH in girls [20]. There are inconsistent results; in most of the studies maternal urinary BPA levels were used. In this paper, we aim to determine the effect of different levels of BPA in cord blood on the thyroid functions, thyroid incretory capacity and thyrotrophic function of the anterior pituitary gland in newborns.

## 2. Material Methods

### 2.1. Study Setting

**Figure 1:**
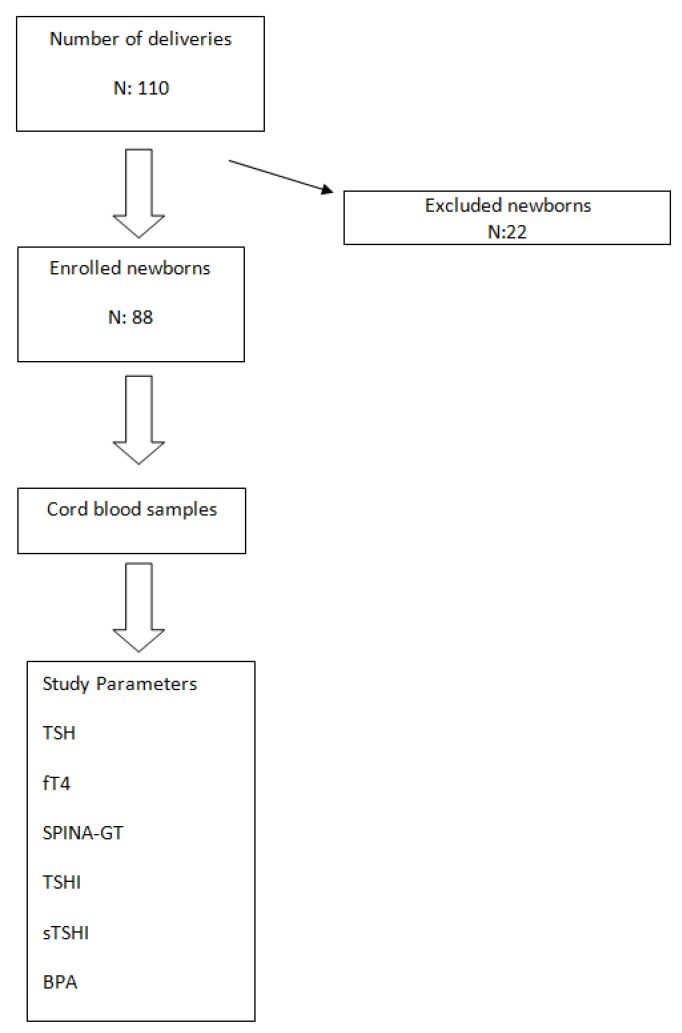
Study Flow Chart.

This study was designed as a cross-sectional study. All expectant mothers under the care of the Department of Gynecology and Obstetrics in Near East University Hospital were invited to participate on the day of delivery between October 2016 and June 2017. Newborns, <37 weeks of gestational age, with a history of maternal thyroid dysfunction, congenital abnormality, perinatal asphyxia and parents who refused to participate in the study, were excluded. Newborns were enrolled in the study after obtaining a written informed consent from parents. Cord blood samples were obtained at birth for measurement of TSH, free T4 and BPA (Figure 1). The study protocol was approved by Near East University, Faculty of Medicine, Ethics Committee for the Protection of Human Subjects, before commencement of the study.

### 2.2. Blood Sampling and Measurements

Cord blood samples were obtained at birth. Blood samples were collected into BPA-free polystyrene tubes (BD Diagnostic Preanalytical Systems, BE). Each blood sample was left to coagulate for 30 min, then samples were centrifuged at 2000× *g* for 10 min at room temperature to obtain serum, which was then stored in aliquots in eppendorf vials at −80 °C until analysis. All samples were studied at the end of the study. On the day of analysis, the aliquots were brought to room temperature and thoroughly vortexed before the analysis.

Thyroid stimulating hormone (TSH) and free thyroxine (fT4) were measured by Abbott commercial kits on an Architect i2000sr instrument (Abbott Laboratories, Abbott Park, IL, USA). The maximum amount of thyroxine that the thyroid can produce in a given time unit (thyroid incretory capacity) was estimated with SPINA-GT and thyrotropic function of the anterior pituitary gland was estimated with a TSH index (TSHI) and standardized TSHI) (sTSHI). SPINA-GT, TSHI and sTSHI were calculated based on fT4 and TSH values [22,23,24].

Total serum concentration of Bisphenol A was analyzed by a sandwich enzyme—linked immunosorbent assays (ELISAs) kits (General Bisphenol A(BPA) ELISA kit, MyBioSource, Inc., San Diego, CA, USA).

The methods of measurement were carried out according to the manufacturer instructions. ELISAs were read with a Spectramax M5 (Molecular Devices, Sunnyvale, CA, USA). The standard curves are created by reducing the date using computer software (Softmax Pro. 5.2) capable of generating a four parameter logistic (4PL) curve-fit.

### 2.3. Statistical Analysis

Statistical analysis was performed using SPSS version 22 for Macintosh (SPSS Inc., Chicago, IL, USA). The results are expressed as mean and standard deviation of the mean (SD). To determine the relationship between quantiles of BPA and the other continuous variables, the Kruskal-Wallis test was used. A *p* value less than 0.05 was considered statistically significant.

## 3. Results

Among 110 deliveries, 22 patients were excluded due to the exclusion criteria, leaving 88 patients in the study group (Figure 1).

Ten mothers had hypothyroidism, 8 did not give permission for the study and 4 were excluded because of a gestational week <37 weeks. Thirty three (37.5%) of newborns were female and 55 (62.5%) male. Mean gestational week was 38.20 ± 1.8 weeks. Birth weight, height and head circumference were 3207 ± 512 g, 48 ± 2.2 cm and 34.11 ± 1.71 cm, respectively. Mean TSH, free T4 and BPA levels were 4.85 ± 1.73 uIu/mL, 0.95 ± 0.2 ng/dL and 4.93 ± 2.33 ng/mL, respectively.

Demographic and laboratory characteristics of the study participants of different genders are shown in Table 1. No statistically significant difference was detected between BPA levels and gestational week, birth weight, birth height and head circumference between genders. The mean BPA level was higher in the female group, without statistical significance (*p*: 0.22).

The measured BPA levels were subgrouped as quantiles: 20%, 40%, 60%, 80% and above 80% (Table 2) in order to evaluate the effect of different levels of exposure to BPA on TSH, fT4, SPINA-GT, TSHI and sTSHI within boys and girls, separately.

## 4. Discussion

Thyroid hormone is known to be essential for fetal and child growth, brain development, metabolic control, and in normal physiological functions including cardiovascular, reproductive and pulmonary systems [25,26].

In this study, no notable association was detected between neither serum concentrations of T4, nor TSH concentrations and cord blood BPA quantiles in both genders. Additionally, no statistically significant effect of BPA on the thyroid’s peripheral function and the function of the anterior pituitary gland, has been detected.

BPA is a monomer of plastic material that is widely used in daily life, such as in cans and bottles used to conserve food and various drinks. This uncontrolled contamination of dietary ingredients results in the bio-acccumulation of BPA in human plasma and tissues, even in cord blood and fetal tissues [27,28]. Interestingly, serum BPA concentrations have been demonstrated to exert gender differences (being higher in men), which are possibly linked to androgen levels [29]. Therefore, in this study cord blood was used to measure BPA levels in order to detect the impact on baseline level of neonates on thyroid functions.

The effect of BPA on human thyroid functions have not been extensively elucidated yet; however, gender differences have been reported in previous cohort studies [14,20]. Therefore, male and female newborns were evaluated separately in this study.

In an experimental study it has been demonstrated that BPA dose-dependently suppreses T3- mediated gene activation, through thyroid receptor alpfa-1 (TRalpfa1) and TRbeta. Additionally, expression of T3-suppressed genes was upregulated by BPA. By that way, BPA acted as an antagonist to T3. This caused a thyroid hormone resistance-like syndrome [30]. Another experimental study revealed that BPA suppressed the CRF-inducible release of TSH with no effect on basal release of TSH from the pituitary gland. They demonstrated that there was no antagonism between TH and BPA in affecting the release of TSH caused by CRF; however, an additive effect was exerted. Moreover, that study demonstrated that relatively high concentrations of BPA had a significant suppressive effect on TRH-inducible and CRF-inducible release of TSH [31]. In another experimental study, Zoeller et al. [32] observed that exposure of rats to BPA during pregnancy and lactation caused an increase in T4 with no effect on TSH on day 15. The authors claimed that there was a balance between the antagonist effect of BPA on the pituitary gland, which would tend to increase TSH release by inhibiting the negative feedback. Meanwhile, the elevated T4 levels in serum would successively tend to suppress TSH release [32].

Results of those experimental studies show that the effect of BPA on thyroid hormones may be dose dependent and is complicated interacting with the dynamics of negative feedback hormone secretion. Therefore, the cord blood BPA levels in our cohort were stratified to quantiles as 20%, 40%, 60%, 80%, and above 80% in order to detect the effect on TSH and fT4 at different concentrations of exposure to BPA.

Results of human studies are conflicting. In a cohort study evaluating maternal BPA level and thyroid functions in newborns; males were found to have reduced TSH levels with higher exposure to BPA [14]. In another study, BPA exposure was found to be associated with lower cord TSH levels in girls [20]; while males demonstrated increased cord blood TSH levels with increasing BPA exposure. Minatoya et al. (cord blood-thyroid hormone newborn) demonstrated no relation between cord blood BPA and thyroid functions in newborns [33]. In the current study no significant effect of BPA on different quantiles has been demonstrated in both genders. Sex-specific effects of BPA on other endocrinological issues has also been reported in several studies. Prenatal BPA exposure in humans was reported to cause sex-specific dysregulation of hypothalamic-pituitary-adrenal axis functions. Higher maternal BPA was associated with an increase in baseline cortisol among girls and a decrease in boys. In contrast, higher BPA exposure resulted in increased reactivity in males but decreased reactivity in females [15]. In a recent study, anogenital distance was demonstrated to be reduced in males with high prenatal BPA exposure [34]. In contrast, we could not detect any effect of BPA on the thyrothrophic function of the anterior pituitary gland.

Mean BPA level was 4934 ± 2332 ng/mL in this study, being higher than studies reported up to date [33,35]. It has been demonstrated that exposure of BPA-containing products to increased temperature results in increased BPA efflux to ingredients. Cyprus has one of the warmest climates in the Mediterranean region, this might have contributed to increased BPA contamination.

One of the limitations of the present study is the sample size that was modest. Studies performed in larger study groups are required to clarify the net effect of BPA on thyroid functions in newborns. Another limitation is that free T3 concentrations have not been analyzed in this study.

In conclusion, although BPA has been demonstrated to contaminate cord blood, no effect on fT4, TSH and the peripheral functions of the thyroid and pituitary gland was detected in our study group. Results of different studies are still conflicting; further studies are warranted.

## Figures and Tables

**Table 1:** Demographic and laboratory characteristics of the study participants.

	Male (*n*: 55) Mean ± SD	Female (*n*: 33) Mean ± SD	*p* Value
Gestational week	38.07 ± 2.2	38.47 ± 0.83	0.135
Birth weight (g)	3283 ± 537	3105 ± 440	0.647
Birth height (cm)	48.51 ± 2.34	48.05 ± 1.88	0.558
Head circumference (cm)	34.39 ± 1.77	33.70 ± 1.59	0.06
TSH (uIu/mL)	4.87 ± 1.83	4.80 ± 1.35	0.05
fT4 (ng/dL)	0.92 ± 0.22	0.97 ± 1.92	0.89
BPA level (ng/mL)	4.51 ± 2.06	5.48 ± 2.56	0.22

**Table 2:** BPA quantiles of all participants.

BPA Quantile	BPA Level (ng/mL)
<20%	0–2.68
20–40% 25	2.64–3.81
40–60% 50	3.81–5.02
60–80% 75	5.02–7.00
>80% 90	>7.00

No statistically significant differences were detected between TSH, fT4, SPINA-GT, TSH and sTSHI according to different quantiles of BPA of the whole study group (data not shown). There was no statistically significant difference in TSH, fT4, SPINA-GT, TSH, sTSHI levels in neither the boys nor the girls based on comparisons of different BPA quantiles (Table 3 and Table 4).

**Table 3:** Results of girls according to BPA quantiles.

	BPA Quantiles					*p* Value
	<20%	20–40%	40–60%	60–80%	>80%	
fT4 (mr*)	13.00	17.00	17.08	19.58	16.60	0.916
TSH (mr*)	20.83	15.25	23.33	14.92	14.70	0.386
SPINA-GT (mr*)	10.00	20.19	13.33	18.17	18.05	0.476
TSHI (mr*)	20.33	15.25	23.17	15.33	14.70	0.433
sTSHI (mr*)	20.33	15.25	23.17	15.33	14.70	0.433

mr*: mean rank.

**Table 4:** Results of boys according to BPA quantiles.

	BPA Quantiles					*p* Value
	<20%	20–40%	40–60%	60–80%	>80%	
fT4 (mr*)	21.88	23.78	31.50	29.59	23.79	0.476
TSH (mr*)	25.08	26.33	23.92	28.18	31.29	0.859
SPINA-GT (mr*)	24.71	26.11	32.00	26.68	19.57	0.50
TSHI (mr*)	23.92	26.22	24.62	28.73	31.29	0.829
sTSHI (mr*)	23.92	26.22	24.62	28.73	31.29	0.829

mr*: mean rank.

## References

[1] Lewis J.B., Rueggeberg F.A., Lapp C.A., Ergle J.W., Schuster G.S. (1999). Identification and characterization of estrogen-like components in commercial resin-based dental restorative materials. Clin. Oral Investig..

[2] Gayathri N., Dhanya C., Indu A., Kurup P. (2004). Changes in some hormones by low doses of di (2-ethyl hexyl) phthalate (DEHP), a commonly used plasticizer in PVC blood storage bags & medical tubing. Indian J. Med. Res..

[3] Guo Y., Zhang Z., Liu L., Li Y., Ren N., Kannan K. (2012). Occurrence and profiles of phthalates in foodstuffs from China and their implications for human exposure. J. Agric. Food Chem..

[4] Kang J.H., Kondo F. (2003). Determination of bisphenol A in milk and dairy products by high-performance liquid chromatography with fluorescence detection. J. Food Prot..

[5] Thayer K.A., Doerge D.R., Hunt D., Schurman S.H., Twaddle N.C., Churchwell M.I., Garantziotis S., Kissling G.E., Easterling M.R., Bucher J.R. (2015). Pharmacokinetics of bisphenol A in humans following a single oral administration. Environ. Int..

[6] Fromme H., Gruber L., Schlummer M., Wolz G., Böhmer S., Angerer J., Mayer R., Liebl B., Bolte G. (2007). Intake of phthalates and di(2-ethylhexyl)adipate: Results of the Integrated Exposure Assessment Survey based on duplicate diet samples and biomonitoring data. Environ. Int..

[7] Sathyanarayana S., Karr C.J., Lozano P., Brown E., Calafat A.M., Liu F., Swan S.H. (2008). Baby care products: Possible sources of infant phthalate exposure. Pediatrics.

[8] Rochester J.R. (2013). Bisphenol A and human health: A review of the literature. Reprod. Toxicol..

[9] Leung A.M., Korevaar T.I., Peeters R.P., Zoeller R.T., Köhrle J., Duntas L.H., Brent G.A., Demeneix B.A. (2016). Exposure to thyroid-disrupting chemicals: A transatlantic call for action. Thyroid.

[10] Muhamad M.S., Salim M.R., Lau W.J., Yusop Z. (2016). A review on bisphenol A occurrences, health effects and treatment process via membrane technology for drinking water. Environ. Sci. Pollut. Res. Int..

[11] Wei J., Lin Y., Li Y., Ying C., Chen J., Song L., Zhou Z., Lv Z., Xia W., Chen X. (2011). Perinatal exposure to bisphenol A at reference dose predisposes offspring to metabolic syndrome in adult rats on a high-fat diet. J. Endocrinol..

[12] Behnia F., Peltier M., Getahun D., Watson C., Saade G., Menon R. (2016). High bisphenol A (BPA) concentration in the maternal, but not fetal, compartment increases the risk of spontaneous preterm delivery. J. Matern. Fetal Neonatal Med..

[13] Poimenova A., Markaki E., Rahiotis C., Kitraki E. (2010). Corticosterone-regulated actions in the rat brain are affected by perinatal exposure to low dose of bisphenol A. J. Neurosci..

[14] Chevrier J., Gunier R.B., Bradman A., Holland N.T., Calafat A.M., Eskenazi B., Harley K.G. (2013). Maternal urinary bisphenol a during pregnancy and maternal and neonatal thyroid function in the CHAMACOS study. Environ. Health Perspect..

[15] Giesbrecht G.F., Ejaredar M., Liu J., Thomas J., Letourneau N., Campbell T., Martin J.W., Dewey D. (2017). Prenatal bisphenol a exposure and dysregulation of infant hypothalamic-pituitary-adrenal axis function: Findings from the APrON cohort study. Environ. Health.

[16] Franssen D., Gérard A., Hennuy B., Donneau A.F., Bourguignon J.P., Parent A.S. (2016). Delayed neuroendocrine sexual maturation in female rats after a very low dose of Bisphenol A through altered GABAergic neurotransmission and opposing effects of a high dose. J. Endocrinol..

[17] Soriano S., Ripoll C., Alonso-Magdalena P., Fuentes E., Quesada I., Nadal A., Martinez-Pinna J. (2016). Effects of bisphenol A on ion channels: Experimental evidence and molecular mechanisms. Steroids.

[18] Boas M., Feldt-Rasmussen U., Main K.M. (2012). Thyroid effects of endocrine disrupting chemicals. Mol. Cell. Endocrinol..

[19] Henrichs J., Ghassabian A., Peeters R.P., Tiemeier H. (2013). Maternal hypothyroxinemia and effects on cognitive functioning in childhood: How and why?. Clin. Endocrinol..

[20] Romano M.E., Webster G.M., Vuong A.M., Zoeller R.T., Chen A., Hoofnagle A.N., Calafat A.M., Karagas M.R., Yolton K., Lanphear B.P. (2015). Gestational urinary bisphenol A and maternal and newborn thyroid hormone concentrations: The HOME Study. Environ. Res..

[21] Ahmed R.G. (2016). Maternal bisphenol A alters fetal endocrine system: Thyroid adipokine dysfunction. Food Chem. Toxicol..

[22] Dietrich J.W., Landgrafe-Mende G., Wiora E., Chatzitomaris A., Klein H.H., Midgley J.E., Hoermann R. (2016). Calculated parameters of Thyroid Homeostasis: Emerging Tools for Differential Diagnosis and Clinical Research. Front. Endocrinol..

[23] Dietrich J.W., Fischer M.R., Jauch J., Pantke E., Gärtner R., Pickardt C.R. (1999). SPINA-THYR: A Novel Systems Theoretic Approach to Determine the Secretion Capacity of the Thyroid Gland. Eur. J. Intern. Med..

[24] Jostel A., Ryder W.D., Shalet S.M. (2009). The use of thyroid function tests in the diagnosis of hypopituitarism: Definition and evaluation of the TSH Index. Clin. Endocrinol..

[25] Diamanti-Kandarakis E., Bourguignon J.P., Giudice L.C., Hauser R., Prins G.S., Soto A.M., Zoeller R.T., Gore A.C. (2009). Endocrine-disrupting chemicals: An Endocrine Society scientific statement. Endocr. Rev..

[26] Miller M.D., Crofton K.M., Rice D.C., Zoeller R.T. (2009). Thyroid-disrupting chemicals: Interpreting upstream biomarkers of adverse outcomes. Environ. Health Perspect..

[27] Mori C. Fetal exposure to endocrine disrupting chemicals (EDCs) and possible effects of EDCs on the male reproductive system in Japan. Proceedings of the International Symposium on Environmental Endocrine Disrupters.

[28] Sakurai K., Mori C. (2000). Fetal exposure to endocrine disruptors. Nippon Rinsho.

[29] Takeuchi T., Tsutsumi O. (1991). Serum bisphenol concentrations showed gender differences, possibly linked to androgen levels. Biochem. Biophys. Res. Commun..

[30] Moriyama K., Tagami T., Akamizu T., Usui T., Saijo M., Kanamoto N., Hataya Y., Shimatsu A., Kuzuya H., Nakao K. (2002). Thyroid hormone action is disrupted by bisphenol A as an antagonist. J. Clin. Endocrinol. Metab..

[31] Kaneko M., Okada R., Yamamoto K., Nakamura M., Mosconi G., Polzonetti-Magni A.M., Kikuyama S. (2008). Bisphenol A acts differently from and independently of thyroid hormone in suppressing thyrotropin release from the bullfrog pituitary. Gen. Comp. Endocrinol..

[32] Zoeller R.T., Bansal R., Parris C. (2005). Bisphenol-A, an environmental contaminant that acts as a thyroid hormone receptor antagonist in vitro, increases serum thyroxine and alters RC3/neurogranin expression in the developing rat brain. Endocrinology.

[33] Minatoya M., Sasaki S., Araki A., Miyashita C., Itoh S., Yamamoto J., Matsumura T., Mitsui T., Moriya K., Cho K. (2017). Cord Blood Bisphenol A Levels and Reproductive and Thyroid Hormone Levels of Neonates: The Hokkaido Study on Environment and Children’s Health. Epidemiology.

[34] Mammadov E., Uncu M., Dalkan C. High prenatal exposure to bisphenol A reduces anogenital distance in healthy male newborns. J. Clin. Res. Pediatr. Endocrinol..

[35] Fenichel P., Dechaux H., Harthe C., Gal J., Ferrari P., Pacini P., Wagner-Mahler K., Pugeat M., Brucker-Davis F. (2012). Unconjugated bisphenol A cord blood levels in boys with descended or undescended testes. Hum. Reprod..

